# Patient and Professional Experiences With Virtual Antenatal Clinics During the COVID-19 Pandemic in a UK Tertiary Obstetric Hospital: Questionnaire Study

**DOI:** 10.2196/25549

**Published:** 2021-08-31

**Authors:** Lauren Marie Quinn, Oluwafumbi Olajide, Marsha Green, Hazem Sayed, Humera Ansar

**Affiliations:** 1 University Hospitals of Leicester Leicester United Kingdom; 2 Department of Obstetrics University Hospitals of Leicester Leicester United Kingdom

**Keywords:** antenatal, virtual clinic, technology, COVID-19, United Kingdom, pandemic, feasibility, effective, telehealth, virtual health

## Abstract

**Background:**

The COVID-19 pandemic required rapid implementation of virtual antenatal care to keep pregnant women safe. This transition from face-to-face usual care had to be embraced by patients and professionals alike.

**Objective:**

We evaluated patients’ and professionals’ experiences with virtual antenatal clinic appointments during the COVID-19 pandemic to determine satisfaction and inquire into the safety and quality of care received.

**Methods:**

A total of 148 women who attended a virtual antenatal clinic appointment at our UK tertiary obstetric care center over a 2-week period provided feedback (n=92, 62% response rate). A further 37 health care professionals (HCPs) delivering care in the virtual antenatal clinics participated in another questionnaire study (37/45, 82% response rate).

**Results:**

We showed that women were highly satisfied with the virtual clinics, with 86% (127/148) rating their experience as good or very good, and this was not associated with any statistically significant differences in age (*P*=.23), ethnicity (*P*=.95), number of previous births (*P*=.65), or pregnancy losses (*P*=.94). Even though 56% (83/148) preferred face-to-face appointments, 44% (65/148) either expressed no preference or preferred virtual, and these preferences were not associated with significant differences in patient demographics. For HCPs, 67% (18/27) rated their experience of virtual clinics as good or very good, 78% (21/27) described their experience as the same or better than face-to-face clinics, 15% (4/27) preferred virtual clinics, and 44% (12/27) had no preference. Importantly, 67% (18/27) found it easy or very easy to adapt to virtual clinics. Over 90% of HCPs agreed virtual clinics should be implemented long-term.

**Conclusions:**

Our study demonstrates high satisfaction with telephone antenatal clinics during the pandemic, which supports the transition toward widespread digitalization of antenatal care suited to 21st-century patients and professionals.

## Introduction

The COVID-19 pandemic presented challenges to obstetric departments worldwide, resulting in increased pressures on the delivery of routine antenatal care. Consensus guidelines recommended that pregnant women should self-isolate and avoid coming into the hospital unless necessary to avoid contracting COVID-19 [1-3]. Following the pandemic, the Royal College of Obstetricians and Gynaecologists recommended a minimum of six antenatal appointments (a reduction from the usual eight face-to-face visits). However, this only accounted for low-risk pregnancies, as higher risk women still required specialist antenatal clinic appointments. Royal College of Obstetricians and Gynaecologists therefore also advised that telephone or video consultations were safe, and their adoption should be maximized to minimize unnecessary contact between patients and care providers [4,5].

Face-to-face antenatal care has long been recognized as the standard of care because of the importance of routine screening for blood pressure and urine assessment, and to provide personalized support. However, telemedicine approaches in perinatal care have been implemented with success [6], and the National Health Service (NHS) England’s long-term plan is a digital agenda aiming to reduce face-to-face appointments by a third over the next 5 years [7]. Pflugieson and Mou [8] compared virtual and traditional antenatal appointments and showed that patient satisfaction was significantly higher in women who received virtual care compared to face-to-face appointments, and there was no difference in preference between telephone or video calls for the virtual clinics. However, during the COVID-19 pandemic, when the majority of antenatal care was rapidly transformed to virtual care in the United Kingdom, it was unclear how pregnant women felt about this shift in culture.

In addition to the patient experience of virtual clinics, it is also important to consider the clinicians’ perspective. A mixed-methods acceptability study evaluating virtual clinics at the micro, meso, and macro levels found that technical challenges could be prohibitive to staff running virtual clinics [9]. However, when clinical, technical, and practical requirements were achieved, staff were satisfied and felt virtual clinics were safe [9]. Nevertheless, in the setting of a global pandemic, virtual clinics had to become the new normality for antenatal care, providing the advantage of instilling digitalized antenatal care into obstetric care systems worldwide [10].

The aim of this study is to evaluate patient and health care professional (HCP) satisfaction, preferences, and experiences of a virtual antenatal clinic during the COVID-19 pandemic from a tertiary obstetric hospital in the United Kingdom. This was important to understand more representative viewpoints from pregnant women when virtual care was the default model of care due to the pandemic, as opposed to virtual care being made available as an option instead of or in addition to traditional face-to-face care as previous studies have explored.

## Methods

### Study Design

In March 2020, antenatal face-to-face general and subspecialist clinic appointments in our regional tertiary obstetric unit were transformed to virtual telephone clinic appointments. Telephone consultations were conducted by either consultants, registrars and junior obstetric doctors, or midwives. The junior obstetric clinic team members discussed all cases with the consultant lead during or at the end of the virtual clinic, and treatment decisions were signed off by the consultant, for example, confirmed mode of delivery and intrapartum care plans (this meant some women may have received more than one call to confirm management plan). The process of setting up the virtual antenatal clinic at our center is reported elsewhere [11].

### Patient Experience

An anonymized questionnaire was used to evaluate satisfaction with the virtual clinic experience. The questionnaire was adapted, with consent, from the questionnaire validated by Pflugieson and Mou [8] (Multimedia Appendix 1). Women rated each question on a Likert scale, from very poor to very good, in relation to scheduling, technology, HCP rating, patient-orientated nature, overall rating, and preferences. There were 16 Likert scale questions. Study participants were finally asked to outline the benefits of the virtual clinic and areas of improvement by free text. The following demographic variables were collected: age, ethnicity, number of previous births, and number of pregnancy losses (see Multimedia Appendix 1 for the patient questionnaire). In light of the pandemic, blood pressure and doppler assessments were organized and performed by the community midwives, as it was not deemed possible to train or deliver this equipment to patients safely.

Women were verbally consented to participate in the questionnaire study during the telephone consultation and could provide feedback by email or telephone questionnaire within 2 weeks of their clinic appointment. A pilot study (n=4 women) was performed to test the questionnaire and acceptability of the study design, and the questions were modified as a result, but this data was not included for analysis.

The questionnaires were collected from women who had a virtual clinic consultation over a 2-week period between Monday, May 4 and Friday, May 15, 2020. This period was chosen because the virtual clinics had been running and optimized over a month prior to this. All questionnaire responses were anonymized, and the feedback was collected independently from the clinic staff. For non-English speaking women, a staff member who could speak the required language (Gujurati, Hindi, or Urdu) obtained feedback by translating over the telephone.

### Health Care Professional Experience

To understand HCPs’ experiences of virtual antenatal clinics, two surveys were produced: the first aimed at clinical staff conducting the virtual antenatal clinics (doctors and midwives: HCP survey) and the second aimed at clerical staff organizing the virtual clinics (administrator survey). The HCP survey included 46 questions, ranked on a Likert scale regarding how safe, effective, efficient, and satisfied HCPs were with the virtual clinics and how this compared to their experience of face-to-face clinics. There were also questions enquiring into the benefits and consequences of virtual clinics and their opinions on how well the virtual clinics had been implemented at this center. Finally, HCPs were asked to give their preference for virtual or face-to-face appointments.

The administrator survey consisted of 26 questions exploring experiences of coordinating the virtual clinics, how this compared to face-to-face clinics, and whether they felt the virtual clinic system was effective.

HCPs were surveyed over a 2-week period in July 2020 and were asked to comment on their experiences from the preceding 3 months. This later time period was chosen because from April to mid-June the obstetric clinic team consisted of 15 individuals who were nonpatient facing but who could undertake the clinics full time. However, from June onwards, the frontline clinical staff started to come back to work in the virtual clinic environment. Therefore, to increase the sample size, we delayed the HCP and administrator surveys to July, when more staff had experienced the new virtual clinic environment, which they could compare to the traditional face-to-face clinics. Importantly, the running of the virtual clinics did not change between the patient survey and the professional survey.

The two surveys were tested on a pilot population of 4 individuals, and the questionnaires were adapted accordingly. A list of all the HCPs and administrators who had undertaken virtual obstetric clinics in the preceding 12 weeks was compiled by the study team (n=45). The questionnaire was sent out via email using Survey Monkey. All questionnaire responses were anonymous (see Multimedia Appendix 2 for the HCP questionnaire).

### Ethics

This study was registered as a service evaluation and quality improvement project with the local audit department (reference number: 10560a).

### Statistical Analysis

Descriptive statistics were performed in Excel (Microsoft Corporation). The Mann-Whitney *U* and chi-square tests were used to compare demographics between responses to the virtual clinic experience. Statistical analysis was performed in Graphpad Prism (GraphPad Software, Inc) and SPSS v22 (IBM Corp). The level of statistical significance was set at *P*<.05.

## Results

### Patients’ Experience of Virtual Antenatal Clinics

In the 2-week period evaluated, 268 women had a virtual consultation and a further 45 women did not attend or cancelled their virtual clinic appointment. Of the 268 women, 28 did not consent to participate in the study. Of the 240 women who were seen in the virtual clinic and consented to providing feedback, 148 completed and returned the questionnaire, resulting in a 62% response rate (see flowchart in Multimedia Appendix 3).

#### Demographics

Of those women who completed the questionnaire, the mean age was 31 (SD 5.829) years. The majority of women were Caucasian (168/148, 78.5%), 20% (30/148) were South Asian, and 1.5% (2/148) were another ethnic group. The majority of women were multipara (132/148, 89.5%), and 10.5% (16/148) of women were primipara. In addition, the majority of women had no previous pregnancy loss (99/148, 67%), and 33% (50/148) had one or more self-reported previous pregnancy losses (miscarriage, ectopic, or molar pregnancy).

#### Quantitative Analysis: Descriptive

**Figure 1:**
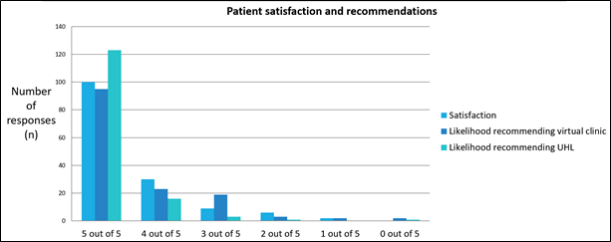
Patient satisfaction and likelihood of recommending virtual antenatal clinics. UHL: University Hospitals of Leicester.

**Figure 2:**
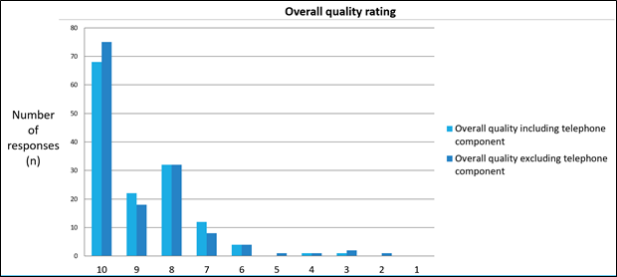
Patient denoted overall quality rating for the virtual antenatal clinics, including and excluding technology elements.

Satisfaction with the virtual clinic appointment was rated as good or very good by 86% (127/148) of women (Figure 1), and 82% (112/148) were very likely or likely to recommend a virtual clinic appointment based on their experience (Figure 1). In addition, 96% (142/148) of women rated the overall quality of the virtual clinic appointment, excluding the technology, component as 6 out of 10 or higher (Figure 2).

Table 1 shows the questions asked in the study questionnaire about their experience of the virtual clinic (adapted from Pflugieson and Mou [8]) and the mean score out of five with the SD provided.

Of those surveyed, 38% (56/148) remembered being asked COVID-19 screening questions, and 12% (18/148) could not remember being asked, leaving 50% (74/148) who were not asked COVID-19 screening questions during the virtual clinic appointment. Almost all women (144/148, 97%) said the consultation felt private to them. When participants were asked to select between virtual and face-to-face appointments, 25% (37/148) preferred virtual, 10% (15/148) had no preference, and 9% (13/148) said it was dependent on other factors such as the ongoing pandemic, giving a total of 44% (65/148) of women who would be happy with virtual appointments. However, 56% (83/148) of respondents still preferred face-to-face appointments.

**Table 1:** Questions asked in the questionnaire with the mean and SD for the Likert score for each rating.

Question number	Question	Likert rating out of 5, mean (SD)
1	Ease of scheduling your virtual clinic appointment	4.5 (0.8)
2	Convenience of virtual clinic times and dates	4.6 (0.8)
3	Ease of connecting for your virtual appointments	4.8 (0.5)
4	Quality of connection during virtual appointments	4.8 (0.5)
5	How well the doctor explained her role in your care	4.4 (1.0)
6	Friendliness/courtesy of doctor	4.8 (0.7)
7	Explanation of plan for next appointment(s) and follow up	4.6 (0.7)
8	Skill and knowledge of the doctor	4.7 (0.7)
9	Degree to which the doctor took time to listen to you	4.6 (0.9)
10	Degree to which doctor helped you to make informed decisions	4.4 (1.0)
11	Doctor’s concern for and ability to answer your questions and worries	4.5 (1.0)
12	Satisfaction with virtual appointments	4.5 (0.9)
13	Likelihood of recommending virtual appointments/your prenatal care doctor	4.4 (1.0)
14	Likelihood that you will continue to seek care from the University Hospitals of Leicester	4.8 (0.6)
15	Overall quality, inclusive of the technology element on a scale of 1-10	8.9 (1.3)
16	Overall quality, exclusive of the technology element on a scale of 1-10	8.9 (1.5)

#### Statistical Analysis

There were no statistically significant differences between virtual clinic satisfaction rating (out of five) and age (*P*=.23), ethnicity (*P*=.95), parity (primipara or multiparous; *P*=.65), or number of previous pregnancy losses (none or ≥1; *P*=.94). There were also no statistically significant differences between preference for clinic type (ie, virtual or face-to-face clinic) with age (*P*=.07), ethnicity (*P*=.93), smoking status (*P*=.78), parity (primipara or multiparous; *P*=.79), or number of previous pregnancy losses (none or ≥1; *P*=.54). When ranking the overall quality of the virtual clinic including the technology components, primipara women were significantly more likely to rate it as 10 out of 10 compared to multiparous women (10/13, 78% vs 59/115, 51%; *P*=.03), whereas multiparous women were significantly more likely to rate it as 7 to 9 out of 10 compared to primiparous women (48/115, 42% vs 2/13, 15%; *P*=.03).

#### Summary of Free-Text Responses

The key benefits highlighted were convenience, avoiding travel, being able to stay at home, and staying safe. Patients also said that the communication from their doctor was good, and the doctors took time to listen to them, were friendly, and gave good explanations. A few women expressed initial concern about the concept of a virtual clinic but were happy with the experience once they had tried it.

In terms of areas for improvement, a lot of women said they would not change anything. However, the main improvement suggested was that the virtual clinic consultations should be at the specific time given. Some women felt they should have been given a choice whether they wanted a face-to-face or virtual appointment.

Of the minority of women who were dissatisfied with their virtual clinic consultation, rating the consultation as very poor or poor, the main issues raised were around the doctor not knowing them and their history well, and wanting a face-to-face appointment because of their individual circumstances, for example, to physically examine them. Timing of the clinic appointment was also a common issue, in terms of having their telephone consultation later than the expected time.

### Health Care Professionals’ Experience of Virtual Antenatal Clinics

#### Demographics

A total of 37 staff members completed the questionnaires, of which 27 completed the HCP survey and 10 completed the administrators survey; this was an 82% response rate overall of the 45 staff members who had performed virtual obstetric clinic duties in the time period evaluated.

Of the 27 HCPs who completed the HCP survey, 38% (n=10) were consultants, 38% (n=10) were registrars, 16% (n=4) were junior doctors, and 4% (n=1) were midwives. In terms of obstetric experience, 33% (n=9) had more than 21 years of experience, 19% (n=5) had 11 to 20 years of experience, and 26% (n=7) had less than 10 years of obstetric experience. Over half of the HCPs (n=15, 54%) had done more than 30 virtual consultations in the study period, 11% (n=3) had done 11 to 30 virtual consultations, and 23% (n=6) had done fewer than 10 consultations.

#### Quantitative Analysis: Descriptive

In terms of HCPs’ experience of the virtual antenatal clinics, 67% (n=18) had a good or very good experience of virtual clinics and 78% (n=21) described their experience as the same or better than face-to-face clinics. Although 74% (n=20) of clinicians received no training, 67% (n=18) felt it was very easy or easy to adapt to the virtual clinics. Nonetheless, 56% felt training would have been helpful or very helpful. A total 78% (n=21) of participants felt the quality of connection was good or very good during the virtual clinics.

The majority felt virtual clinics were safe (n=22, 82%), 100% (n=27) felt virtual clinics were effective at delivering on high quality patient care, and 89% (n=24) perceived the care received by patients to be better or comparable to face-to-face appointments. Furthermore, 56% (n=15) felt it was easier or just as easy to seek advice or a second opinion in virtual clinics.

Although 74% (n=20) perceived virtual clinics took longer than face-to-face appointments, 63% (n=17) felt virtual clinics were as or more efficient than face-to-face clinics overall. Within each 4- to 4.5-hour clinic period, 70% (n=19) of HCPs could review on average 5 to 8 patients in a virtual clinic, while 22% (n=6) could consult with more than 9 patients. Importantly, 93% (n=25) felt virtual clinics should be implemented in the long-term. In terms of clinician preference, 44% (n=12) gave no preference, 15% (n=4) preferred virtual clinics, and 27% (n=7) preferred face-to-face appointments.

In terms of clerical staffs’ experience of coordinating the virtual antenatal clinics (n=10), 70% (n=7) found it easy or very easy to schedule the virtual clinic appointments for patients, but 50% (n=5) felt the virtual clinics took more time to complete the clinic outcomes for patients. All (n=10, 100%) felt the care received by patients was the same or better with the virtual clinics compared to the face-to-face appointments, and 80% (n=8) felt virtual clinics were safe for patients. Of these, 80% (n=8) rated their experience of virtual clinics as good or very good, and 80% (n=8) said the virtual clinics in their current format would be feasible for the future and should be implemented long-term. Finally, 60% (n=6) had no preference for virtual or face-to-face clinics, and 90% (n=9) found it easy or very easy to adapt to the virtual clinics.

HCPs’ perceived benefits of virtual antenatal clinics included patient convenience, environmentally friendly, and cost-effectiveness. Further benefits that ranked highly include the improved efficiency of virtual clinics, staff convenience, and patient-centered approach.

The principal barriers included the unavoidable need for face-to-face appointments in certain cases, limitations with technology, difficulties embedding the virtual clinics into the systems and process, and the adaptation required by clerical and clinical teams alike.

#### Summary of HCP Free-Text Responses

Key themes from the HCPs that arose were that the virtual clinics reduced unnecessary visits to the hospital for patients and allowed low-risk pregnancies to be managed safely from the patient’s own home. Many felt patient compliance was better and perceived fewer *did not attend* appointments, and the clinics were considered to be patient-centered with good continuity of care. A concern raised was around a reduction in the training for junior doctors in virtual clinics compared to face-to-face clinics; however, others felt team cohesion was better, and they were able to discuss more cases with senior colleagues during the virtual clinics.

## Discussion

### Main Findings

We demonstrated that women were highly satisfied with all aspects of their virtual clinic experience and that this did not differ with age, ethnicity, previous pregnancies, or previous pregnancy losses. Although 56% (83/148) of women in our study would prefer a face-to-face clinic appointment, 44% (65/148) preferred virtual clinics or had no preference. We also showed that HCPs and administrative staff in our center were highly satisfied with virtual antenatal clinics and felt they were at least comparable to face-to-face appointments in terms of safety, care received, efficiency, and experience.

The COVID-19 pandemic has provided a much needed opportunity to digitalize antenatal care; the vast majority of pregnant women own a smartphone [12] and commonly seek knowledge from online sources [13]. eHealth has been shown to confer benefits to lifestyle and mental health outcomes for pregnant women, who report that eHealth interventions are convenient and acceptable [14]. Hence, there is scope to increasingly integrate virtual and telemedicine approaches to bring antenatal care into the 21st century [15].

Our finding that virtual antenatal clinics were acceptable to all patients surveyed regardless of sociodemographic differences is supported by Pflugieson and Mou [8] who showed similarly high satisfaction with virtual care. Historically, women’s uptake for virtual care has been limited due to concerns about lack of perceived support and long gaps between in-person visits [16,17]. However, a shift in practice with the pandemic has allowed increased capture of a wider proportion of women’s preferences. Holcomb et al [18] showed high patient satisfaction with audio-only virtual antenatal care during the COVID-19 pandemic, demonstrating that 99% of women felt their needs were met with virtual care, and compliance with virtual clinics (88%) was significantly higher than in-person appointments (82%; *P*<.001). A cross-sectional study by Futterman et al [19] compared virtual with in-person appointments and found high satisfaction with both, although in-person satisfaction was significantly higher. We therefore acknowledge, similar to Pflugieson and Mou [8], that women should ideally be offered a choice in their antenatal care modality because of the unique benefits received from patient-centered, face-to-face contact with a HCP. Aziz et al [6] similarly reported on the importance of combining face-to-face and telemedicine approaches for high-risk pregnancies during the pandemic, but we must ensure adoption of telemedicine strategies that do not compromise materno-fetal outcomes. A randomized controlled trial by Butler Tobah et al [20] compared alternative prenatal care (with fewer on-site visits and more virtual appointments) with usual face-to-face care and showed that women had higher levels of satisfaction and less stress with the virtual care arm, with no difference in materno-fetal outcomes or perceived quality of care. These findings have been further supported by systematic reviews and studies that have confirmed safety and efficacy of virtual clinics and telehealth despite a reduction of in-person visits [21-23]. Where face-to-face appointments cannot be avoided, Dashraath et al [24] outlined how in-person antenatal care can be safely practiced in the context of the COVID-19 pandemic, with social distancing and appropriate personal protective equipment for staff and women alike [24]. However, despite these important measures, Fryer et al [15] acknowledged the further work that needs to be done to protect higher risk pregnancies and ensure health inequality gaps are not widened.

The high professional satisfaction with virtual care reported in our study can be attributed to the simple model of telephone consultations that we adopted, which minimized the technical issues experienced. Greenhalgh et al [9] performed a multilevel analysis of virtual clinics, pre–COVID-19, in which they experienced significant technical issues that were prohibitive to implementation, resulting in a virtual consultation rate of only 22%. Nevertheless, our study is the first, to our knowledge, to evaluate the HCP perspective of virtual clinics during and before the COVID-19 pandemic. We found good integration between clinical and administrative teams to ensure the clinics ran efficiently and meant satisfaction from both teams was high. The finding that our virtual clinics were perceived to take longer than face-to-face appointments is not supported by other studies [9,16] and may be explained by inexperience or apprehension around needing to adapt to the new clinic experience, which is recognized as a significant challenge for HCPs; staff were asked to transition to a completely new way of working without training beforehand [9]. However, given that virtual clinics were considered to be more efficient than face-to-face appointments, this would suggest time was saved elsewhere. The majority of staff surveyed were in support of training for virtual clinics, which is not routinely part of the curriculum, and we anticipate this would further improve efficiency, satisfaction, and ease of adaptation to virtual clinics.

The benefits of virtual clinics are widely reported to include the environmentally friendly nature, patient-centered approach, and opportunity to build better rapport between patient and professionals. Furthermore, the challenges presented such as inability to examine can be overcome with video consultations [25]. Therefore, the benefits combined with the high satisfaction reported here demonstrate the importance of integrating virtual clinics into obstetric care services in the longer term to align with the NHS digital long-term plan [7].

### Strengths and Limitations

We achieved a 62% (92/148) questionnaire response rate for women who received a virtual clinic consultation and 82% (37/45) response rate for the professionals surveyed in a large UK tertiary obstetric care center during the study period. Pflugieson and Mou [8] attained a 12.1% to 19.8% response rate in their study [8], hence reflecting the widely representative nature of our study findings.

Limitations of the study include its cross-sectional nature, as we only evaluated patient satisfaction over a 2-week period, and collection of the feedback was retrospective. There was also risk of selection bias in terms of the population of women who chose to complete the study questionnaire, but the majority response rate (92/148, 62%) will have negated this effect. The HCP sample size (n=37) was small because of the single center nature of this study, but attaining an 82% (37/45) response rate will have provided representative perspectives from this small cohort. Future work should be performed in larger, more diverse cohorts of HCPs and patients to further elucidate our study findings and to better differentiate virtual clinic preferences. We were unable to compare virtual clinic experiences directly with face-to-face care because of the pandemic, but as the lockdown restrictions ease, this is an area we will be evaluating moving forward to assess the long-term feasibility of virtual antenatal clinics. It is important to note that a minority of our cohort were primipara women, and the majority had no previous pregnancy loss, which reflects a lower risk population and may partly account for the high satisfaction with virtual compared to face-to-face consultations. Furthermore, this questionnaire was only designed to evaluate preferences, and further work needs to focus on safety, cost-effectiveness, and maternal and fetal outcomes of virtual care compared to face-to-face antenatal clinics. Finally, it must be acknowledged that satisfaction was not 100%, and for a minority of patients and professionals, virtual care and clinics were not acceptable. In these instances, a combination of virtual and face-to-face care would be a necessary approach, which aligns with the consensus viewpoint for antenatal care services.

### Conclusion

We have shown that, despite rapid transformation and implementation of virtual antenatal clinics during the COVID-19 pandemic, patient and professional satisfaction with this service was very high. The virtual antenatal clinics have been widely accepted by women regardless of sociodemographic differences, which supports feasibility of the virtual clinics moving forward. Our study supports integration of virtual antenatal clinics alongside face-to-face delivery of care as and where appropriate to ensure delivery of patient-centered care. Further telemedicine strategies that aim to personalize care for pregnant women warrant further exploration.

## Appendix

Multimedia Appendix 1 Virtual antenatal clinic survey for pregnant women.

Multimedia Appendix 2 Virtual antenatal clinic survey for professionals.

Multimedia Appendix 3 CONSORT flow diagram.

## Abbreviations

HCP: health care professional
NHS: National Health Service
