# Supplementary material for: Patient and Professional Experiences With Virtual Antenatal Clinics During the COVID-19 Pandemic in a UK Tertiary Obstetric Hospital: Questionnaire Study
Source: J Med Internet Res. 2021 Aug 31;23(8):e25549. doi: 10.2196/25549 (PMC8409501; doi:10.2196/25549)
Supplement: Multimedia Appendix 1 [file jmir_v23i8e25549_app1.docx]

Telephone clinic care survey: For patients

Thankyou for agreeing to participate in this questionnaire study. The feedback you provide will be anonymised and will be invaluable to help us improve the telephone antenatal clinic.

For each of the questions below, please choose from 1 to 5, where 1 is very poor and 5 is very good. Rating scale: 1=very poor, 2=poor, 3=average, 4=good, 5=very good

The questions relate to your experience of your telephone clinic consultation(s) only with a doctor from the University Hospitals of Leicester.

| Question | Rating:  on a scale of 1-5.  Rating scale: 1=very poor, 2=poor, 3=average, 4=good, 5=very good |
| --- | --- |
| Ease of scheduling your virtual clinic appointment: | /5 |
| Convenience of virtual clinic times and dates: | /5 |
| Ease of connecting for your virtual appointments: | /5 |
| Quality of connection during virtual appointments: | /5 |
| Ease of sending scans via email: (if applicable) | /5 |
| Ease of sending scans via whatsapp: (if applicable) | /5 |
| How well the doctor explained her role in your care: | /5 |
| Friendliness/courtesy of doctor | /5 |
| Explanation of plan for next appointment(s) and follow-up | /5 |
| Skill and knowledge of the doctor | /5 |
| Degree to which the doctor took time to listen to you | /5 |
| Degree to which doctor helped you to make informed decisions | /5 |
| Doctor’s concern for  and ability to answer your questions and worries: | /5 |
| Were you asked COVID screening questions? | No |
| Did the consultation feel private? | Yes |
| Satisfaction with Virtual appointments | /5 |
| Likelihood of recommending virtual appointments/your prenatal care doctor | /5 |
| Likelihood that you will continue to seek care from UHL | /5 |
| Did you receive your patient information leaflets as discussed? | No |
| Would you rather have a virtual clinic appointment or a traditional appointment? | virtual |
| Number of telephone clinic appointments? |  |
| Overall quality, inclusive of the technology element  on a scale of 1-10: | /10 |
| Overall quality, exclusive of the technology element on a scale of 1-10: | /10 |
| What was the best part about the virtual clinic appointment? (please outline your reasons) |  |
| How could the virtual clinic be improved? (please outline your answer below) |  |
| Number of previous pregnancies and children? |  |
| Ethnicity |  |
| Age: |  |
| Smoking? Current or ex-smoker |  |
