# Supplementary material for: Patient and Professional Experiences With Virtual Antenatal Clinics During the COVID-19 Pandemic in a UK Tertiary Obstetric Hospital: Questionnaire Study
Source: J Med Internet Res. 2021 Aug 31;23(8):e25549. doi: 10.2196/25549 (PMC8409501; doi:10.2196/25549)
Supplement: Multimedia Appendix 2 [file jmir_v23i8e25549_app2.docx]

Virtual clinic survey-for professionals:

Thankyou for agreeing to participate in this questionnaire study. The feedback you provide will be anonymised and will be invaluable to help us improve the telephone antenatal clinic.

For each of the questions below, please choose from 1 to 5, where 1 is very poor and 5 is very good. Rating scale: 1=very poor, 2=poor, 3=average, 4=good, 5=very good

Please kindly return this to us by Friday 26^th^ June 2020.

| Question | Rating:  on a scale of 1-5.  Rating scale: 1=very poor, 2=poor, 3=average, 4=good, 5=very good |
| --- | --- |
| Ease of scheduling the virtual clinic appointments: | /5 |
| Convenience of virtual clinic times and dates: | /5 |
| Ease of connecting for the virtual appointments: | /5 |
| Quality of connection during virtual appointments: | /5 |
| Ease of getting hold of the patients to book appointments or for the virtual clinic appointment | /5 |
| Ease of receiving scans via email: (if applicable) | /5 |
| Ease of receiving scans via whatsapp: (if applicable) | /5 |
| Average number of patients seen by yourself per clinic (clinician) | Number= |
| How would you personally rate your virtual clinic appointments? | /5 |
| Ease of generating a follow-up plan? | /5 |
| Ease of clinic outcomes being actioned? | /5 |
| Ease of using the clinic proforma? | /5 |
| How useful is the clinic proforma? | /5 |
| Ease of making informed decisions for your patients? | /5 |
| Ease of accessing senior advice or a second opinion? | /5 |
| How would you rate the overall standard virtual care received by your patients? | /5 |
| Patient’s satisfaction with Virtual appointments? | /5 |
| To what extent do you think virtual clinics are safe? | /5 |
| To what extent do you think virtual clinics are effective? | /5 |
| To what extent do you think virtual clinics are feasible? | /5 |
| What proportion of clinics do you think should be virtual clinic | Percentage  0-25%  25-50%  50-75%  75-100% |
| Likelihood of recommending Virtual appointments? | /5 |
| Would you rather conduct a virtual clinic appointment or a traditional appointment? | Virtual or face to face, either/or? |
| Type of virtual clinics conducted? |  |
| Did your virtual clinic consultations feel private to you? | Yes or No |
| Did you receive training for virtual clinics? | Yes or No |
| To what extent would training be helpful for conducting virtual clinics? | /5 |
| Would you prefer video consultation or telephone? | Video or telephone or either/or |
| Number of telephone clinic appointments conducted? | >50  30-50  20-30  10-20  <10 |
| Overall quality, inclusive of the technology element  on a scale of 1-10: | /10 |
| Overall quality, exclusive of the technology element on a scale of 1-10: | /10 |
| What was the best part about the virtual clinic appointment? (please outline your reasons) |  |
| What challenges were presented with the virtual clinic appointments? |  |
| How could the virtual clinic be improved? (please outline your reasons) |  |
| Grade: | Admin  MCA  MW  SHO  Registrar Consultant |
| Years of experience in obstetrics |  |
