# Supplementary figures and images for: Patient and Professional Experiences With Virtual Antenatal Clinics During the COVID-19 Pandemic in a UK Tertiary Obstetric Hospital: Questionnaire Study
Source: J Med Internet Res. 2021 Aug 31;23(8):e25549. doi: 10.2196/25549 (PMC8409501; doi:10.2196/25549)

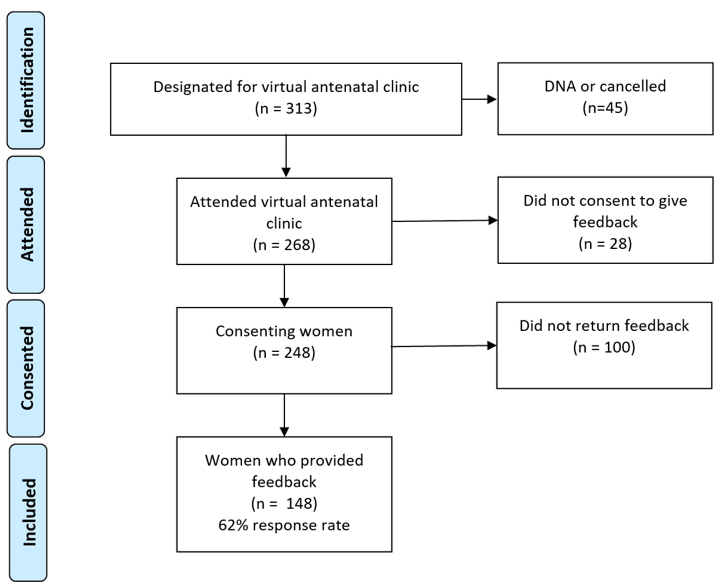

Supplement: Multimedia Appendix 3 [file jmir_v23i8e25549_app3.png]
